# Ultrafast Temporal-Spatial Dynamics of Phase Transition in N-Doped Ge_2_Sb_2_Te_5_ Film Induced by Femtosecond Laser Pulse Irradiation

**DOI:** 10.3390/mi13122168

**Published:** 2022-12-08

**Authors:** Hao Wu, Xiaobin Zhang, Weina Han

**Affiliations:** 1 Laser Micro/Nano-Fabrication Laboratory, School of Mechanical Engineering, Beijing Institute of Technology, Beijing 100081, China; 2 Beijing Institute of Technology Chongqing Innovation Center, Chongqing 401120, China

**Keywords:** nitrogen doped GST, temporal-spatial-resolved dynamics, femtosecond laser excitation, phase transition evolution, a bond effect

## Abstract

Element-doped phase change material (PCM) could improve the performances, e.g., better thermal stability, higher electrical resistance, and faster crystallization speed; thus, the influence of the doping element needs to be further investigated. In this paper, a femtosecond laser, which could realize the ultrafast phase transition rate of PCM between amorphization and crystallization, was used to explore the properties of nitrogen-doped Ge_2_Sb_2_Te_5_ (GST), and a bond effect was proposed. The pure GST and different nitrogen contents of doped GST films were investigated by femtosecond laser pulse excitation through a pump–probe shadowgraph imaging technique. The results showed that the element-doped films could change photon absorption because of the increase in free carriers. This caused the faster rate of reflectivity to change in the irradiated area by the laser beam as the more nitrogen doped. When the nitrogen content increased, the crystallization evolution became harder because it enhanced the bond effect, which suppressed crystalline grain growth and improved the thermal stability. Based on the analysis in the paper, the desired performances of PCMs, e.g., ultrafast dynamics, crystallization evolution, and thermal stability, could be controlled according to the demands by modifying the bond effect.

## 1. Introduction

PCM has attracted a lot of interest for applications, e.g., novel non-volatile memory [1], switches [2], electrically- or optically-driven intelligent displays [3], sensors [4], etc., due to advantages such as fast phase switching speed [5], low-cost consumption [6], good scalability [7], and high endurance [8]. PCM works as a switch by the changes (i.e., electrical optical refractivity and/or resistance) between its amorphous and crystalline states [9]. The ternary Ge-Sb-Te compounds, for example Ge_2_Sb_2_Te_5_ (GST) and others along the pseudo-binary GeTe-Sb_2_Te_3_ tie line, have become promising candidates among PCMs [10] because of the excellent optical and electrical properties, good thermal stability of GeTe, and fast phase change ability of Sb_2_Te_3_ material [11]. 

Nevertheless, to improve the properties of the PCMs, elements such as gas elements (e.g., nitrogen [12]), metallic elements (e.g., nickel [13], zinc [14], and magnesium [15]), and non-metallic elements (e.g., silicon [16]) were doped in the materials. Zhou et al. [17] investigated the carbon-doped Ge_2_Sb_2_Te_5_ and found that the incorporated carbon increased the thermal stability of the PCMs because of the enhanced covalent nature of the material with larger tetrahedral Ge sites. Yao et al. [18] doped nitrogen into Ge_2_Sb_2_Te_5_, and the experimental results showed that not only did the N-doped GST exhibit better thermal stability, but the sheet resistance in the crystalline state improved. Moreover, the Sc-doped Sb_2_Te_3_ material was studied, which could speed up the crystallization speed [19]. 

The mentioned studies on the phase transition properties of PCMs have been reported by means of thermal annealing for several minutes to hours [20]. However, the amorphization process of PCMs typically could occur in tens of nanoseconds, while the crystallization process would be a little slower in about tens to hundreds of nanoseconds because of the required time for nuclei to form and grow [21]. A pulse laser beam can realize a high transition rate between amorphization and crystallization [22]. Furthermore, because of the lots of free carriers excited by the ultrafast laser in GST, the non-equilibrium heat transfer would take place, which had an impact on the amorphization and crystallization [22]. Thus, an ultrafast laser, as a tool, was used to study the thermal stability [23], crystallization mechanism [24], and characterization [11] of the element-doped PCMs. However, the performance and mechanism of the influence of the element-doped PCMs still need to be further studied.

In this paper, the properties of nitrogen-doped GST, as an example of element-doped PCMs, are studied in this paper, which demonstrates the impact of element doping. The ultrafast dynamics of pure GST and different nitrogen contents doped GST induced by a femtosecond laser pulse, one kind of ultrafast laser, are studied by the reflective-type pump-probe experiment, and the Raman spectrum results on doping reveal the crystallization characterization and the crystallization mechanism on laser irradiation regions. In addition, the microstructures on doping are studied by Scanning Electron Microscope (SEM), and the crystallization evolution is investigated by Transmission Electron Microscope (TEM). The results show a bond effect, which is the essential reason for changing the properties when doping. Thus, different performances of PCMs can be derived according to demands by modifying the bond effect.

## 2. Experiment

**Figure 1:**
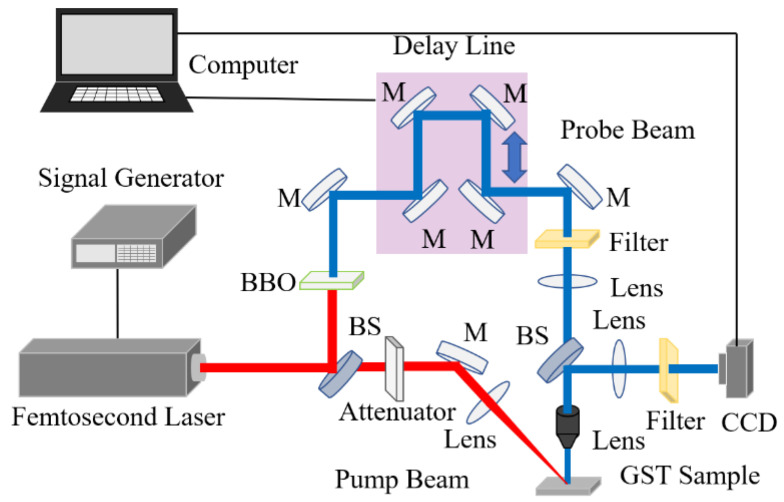
Schematic of the reflective-type pump-probe system.

The nitrogen-doped Ge_2_Sb_2_Te_5_ films were grown on a Si single-crystal wafer by magnetron sputtering from the Ge_2_Sb_2_Te_5_ target (the materials showed a composition of Ge_22.25_Sb_22.32_Te_55.43_ measured by EDS before the experiments, which almost close to the desired stoichiometry) at room temperature [25]. The different nitrogen contents in the films were controlled by changing the ratio of Ar and N_2_ gas flow rates in the range of 2:0–2:2. The ratio of Ar and N_2_ gas flow rates of 2:0 was the amorphous phase state of GST; thus, they were designated as pure GST. The ratios of Ar and N_2_ gas flow rates of 2:1 and 2:2 were designated as 2:1 and 2:2 films, respectively. Ar gas flow rates were fixed at 35 sccm, and these films with a thickness of 100 nm were deposited, which was confirmed by a three-dimensional topography instrument, when the sputtering power was 30 W. Then, the ultrafast dynamics of three kinds of films were studied by reflective-type pump-probe experiments, and the schematic of the temporal-spatial pump-probe system is shown in Figure 1. A femtosecond laser (Spitfire, Spectra-Physics Inc., Milpitas, CA, USA) with a wavelength of 800 nm and a pulse duration of 35 fs was used to generate pulses controlled by a signal generator, which were divided into pump and probe pulses using a beam splitter. As the focal length of the lens, the size of the focused spot, and the angle of incidence did not have an effect on the analysis of the ultrafast dynamics, the pump pulses were focused on the films through a lens with a focal distance of 150 mm at an angle of 45°. The probe pulses were frequency doubles by a beta barium borate crystal and delayed to irradiate the excited region of the films perpendicularly by an optical delay line. There were two lenses used to expand the diameter of the directional probe beam focused on the sample in order to decrease the fluence of the probe beam less than the phase transition threshold to eliminate its influence on the results. The temporal-spatial reflective images were taken by a charge-coupled device (CCD) through a 20× objective (NA = 0.45, Olympus Inc., Tokyo, Japan). In addition, a 400 nm bandpass filter was used to eliminate the impact of the pump pulses and the plasma in front of the CCD. Finally, the morphology of the three kinds of films was studied by SEM, and the phase states of the sample were investigated by TEM and a commercial Raman system.

## 3. Results and Discussion

### 3.1. Spatial-Temporal Resolved Measurement of Femtosecond Laser Pulses Exciting Three Kinds of Films

**Figure 2:**
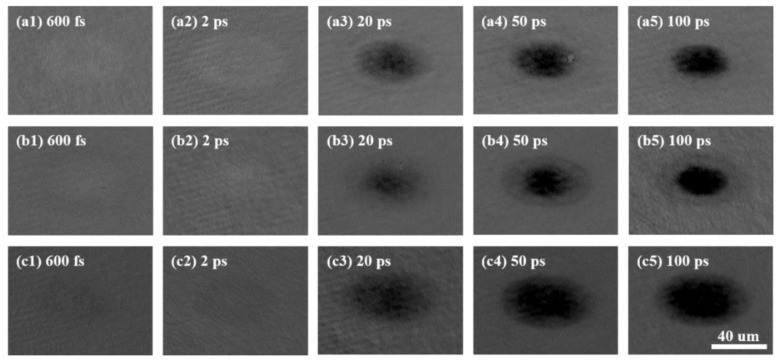
Time-resolved reflectivity of the excited areas on pure GST and different nitrogen content GST films by a single femtosecond laser pulse with a fluence of 0.034 J/cm^2^. (**a1**–**a5**) Images of time-resolved reflectivity of pure GST films, (**b1**–**b5**) Images of time-resolved reflectivity of 2:1 films, (**c1**–**c5**) Images of time-resolved reflectivity of 2:2 films.

To ensure a strong signal-to-noise ratio, a laser fluence of 0.034 J/cm^2^, which is more than all of the films’ ablation thresholds, was selected in reflective-type pump-probe experiments. Figure 2 shows the transient non-equilibrium state of different kinds of GST excited by a single femtosecond laser pulse. The images’ reflectivity of each kind of film is under a series of changes after laser irradiation. It is shown that the area becomes a litter obscure and bright in the sub-picosecond period for the pure GST in Figure 2a1. Then, the size of the irradiated spot grows into an elliptical area, and the center becomes dark gradually in the next picoseconds, as shown in Figure 2a2–a5. Meanwhile, the surroundings also become dark. Similarly, the changes in reflectivity have the same trends for the 2:1 films, which can be found in Figure 2b1–b5. However, with regard to the 2:2 films, the irradiated area is dark at the beginning in Figure 2c1. Then, the size becomes bigger, and the reflectivity becomes darker in this area, as shown in Figure 2c2–c5. For the same laser fluence, the trends of reflectivity are different for the three kinds of GST films. The normalized reflectivity changes dramatically with the increase of the time delay for the 2:2 films, while it changes relatively gently for the pure GST films. 

**Figure 3:**
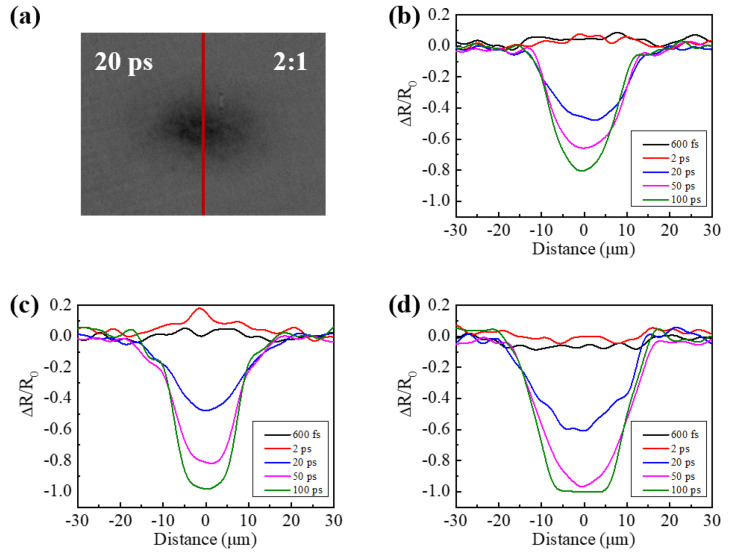
(**a**) The extracted curve of the spatial reflectivity distributions in Figure 2b3 for an example, (**b**) Spatial reflectivity distribution of the pure GST films extracted from the short axis of the focal area in Figure 2a1–a5, (**c**) Spatial reflectivity distribution of 2:1 films extracted from the short axis of the focal area in Figure 2b1–b5, and (**d**) Spatial reflectivity distribution of 2:2 films extracted from the short axis of the focal area in Figure 2c1–c5.

The spatial reflectivity distributions of pure GST and different nitrogen content GST films extracted from the short axis of the elliptical focal area in the images taken by reflective-type pump-probe experiments (for example, the reflectivity of the curve in Figure 3a) are shown in Figure 3b–d for a detailed discussion. When the delay time is from 20 ps to 100 ps, the normalized reflectivity for the three kinds of films presents a Gaussian distribution, except for the time delay of 100 ps for the 2:2 films, which is a flat-top profile. It is meant that the laser beam is absorbed by the 2:2 films strongly and attributed to the superheating of liquid, called the overshooting phenomenon [26]. However, when the delay time is shorter than 20 ps, the curves of the reflectivity for these films have different profiles. The reflectivity of the laser irradiation regions for pure GST films and 2:1 films is above 0 at 600 fs and 2 ps, while that of the 2:2 films is below 0.

**Figure 4:**
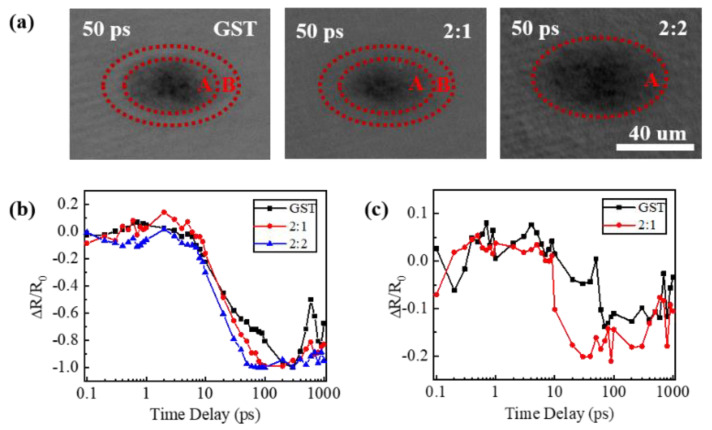
(**a**) Zones A and B in spatial-resolved images of three kinds of films at a time delay of 50 ps after exposure to the pump pulse; (**b**) Reflectivity dynamics of Zone A for the pure GST, 2:1 and 2:2 films; (**c**) Reflectivity dynamics of Zone B for the pure GST and 2:1 films.

To explore the reflectivity dynamics of the films, the reflectivity of the images in Figure 2 can be identified into different zones based on the laser fluence because the intensity of the laser beam yields the Gaussian distribution. As shown in Figure 4a, two zones of “A” and “B” are defined to distinguish the different reflectivity characters in the images of three kinds of GST at a time delay of 50 ps, for example. Zone A represents the center, which is excited by the relatively high laser fluence of the pure GST. Zone B is the area with low reflectivity during transient evolution, where it is irradiated under the relatively low laser fluence. Similarly, there are reflectivity dynamics of Zones A and B for the 2:1 films. However, there is only Zone A for the 2:2 films because the reflectivity gets dark directly according to the value of reflectivity below 0 all the time in Figure 3d. The divided zones for the 2:1 and 2:2 films are also shown in Figure 4a. It can be seen that with the increase of the nitrogen doped, the area of Zone A becomes bigger. It is indicated that when the same fluence irradiates on the films, the reflectivity of the 2:2 films changes the most easily, and that of the pure GST films varies the hardest. This is because nitrogen doping increases the free carriers in the films. In addition, due to the easy changes in reflectivity for the 2:2 films, the laser fluence is high enough to cause the irradiated area to be dark. This is the reason that Zone B does not appear for the 2:2 N-doped GST under this circumstance. 

Furthermore, as the areas of Zones A and B change with the time delay, the average value of the area with 2 × 2 pixels at the center is selected as the reflectivity of Zone A, and the average value of three random points is chosen as the reflectivity of Zone B to study the ultrafast dynamics. Reflectivity of Zones A and B is a function of the delay time after laser excitation on the films, shown in Figure 4b,c, respectively. Although the signal-to-noise is not high, it can be seen that the reflectivity increases slightly when the delay time is from the beginning to about 2 ps in Zone A for pure GST films and 2:1 films. Because the laser fluence is too high according to the analysis above, the reflectivity directly decreases in the first about 1 ps, and then rises a little bit from about 1 ps to about 2 ps for 2:2 films. The 2:1 films reach the highest reflectivity at a delay time of about 2 ps after excitation among the films, while the 2:2 films have the lowest reflectivity. The curves of the three kinds of films show a similar trend from the beginning to about 2 ps. This is because when a single femtosecond laser pulse irradiates the films, the free electrons and holes are excited, and the probe pulse is reflected. It is inferred that when the laser fluence decreases to some degree, the reflectivity of 2:2 films will increase slightly first, which means that the irradiated area can become bright at the beginning, such as other films. When the delay time is from 2 ps to 100 ps, the reflectivity of these films decreases gradually first, and then drops dramatically. This process means that the energy transfers from the free carrier to the lattice of the films. The reflectivity of the pure GST decreases most slowly, while that of the 2:2 films has the fastest reduction. It can be seen that when the delay time is from about 60 ps to 100 ps, the reflectivity of the 2:2 film stays at a value of −1. This means that the probe pulse has been nearly absorbed completely, which corresponds to the analysis of the flat-top curve in Figure 3c. Because the beam fluence in Zone A is more than the phase state changing threshold in the experiment, the crystallizations of these films occur. In addition, the reflectivity dynamics of Zone B irradiated by low fluence versus the delay time are plotted in Figure 4c. The mechanism of the change in reflectivity trends for pure GST and 2:1 films is the same as in the analysis above. With nitrogen doping, the rate of change in reflectivity in the irradiated area by the laser beam can be changed. It is indicated that element doping can increase the free carriers in the films and change the photon absorption; thus, ultrafast dynamics can be controlled by modifying element content in GST.

### 3.2. Morphology Distribution and Crystallization Characterization of the Irradiation Regions for Three Kinds of Films

**Figure 5:**
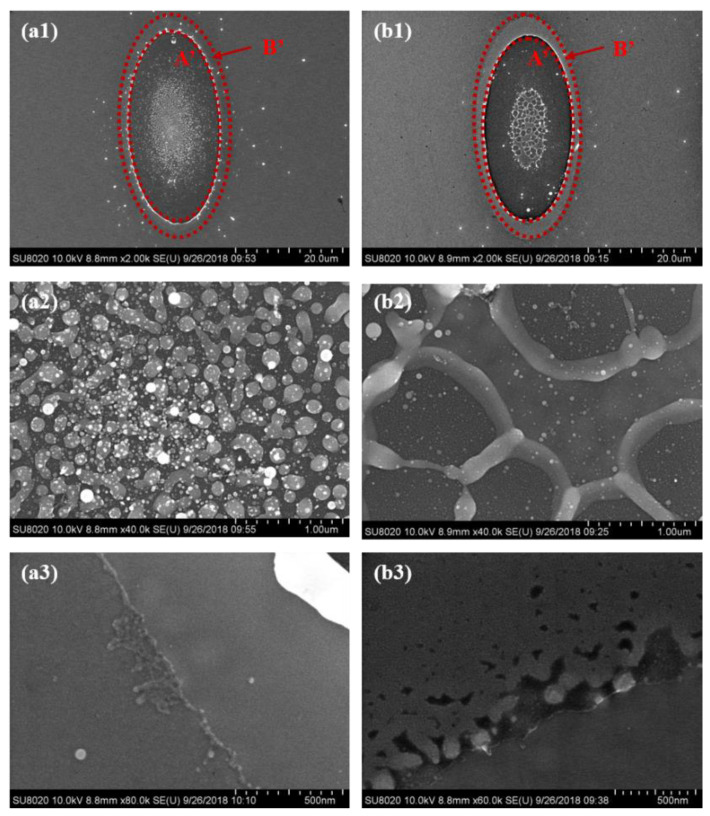
(**a1**) SEM image of the pure GST film after the reflective-type pump-probe experiments, (**a2**) SEM image of the pure GST in Zone A’, (**a3**) SEM image of the pure GST in Zone B’, (**b1**) SEM image of the N-doped GST film after the reflective-type pump-probe experiments, (**b2**) SEM image of the N-doped in Zone A’, and (**b3**) SEM image of the N-doped in Zone B’.

The morphologies of the pure GST and N-doped GST after the reflective-type pump-probe experiments are observed by SEM. As shown in Figure 5a1,b1, the structure of these films after laser irradiation is almost the same. However, the ablated area of the N-doped GST is smaller than that of the pure GST, which means that it is harder to ablate the N-doped GST compared to the pure GST. It is indicated that thermal stability improves after nitrogen is doped. Moreover, two different zones can be distinguished. Zone A’ represents the manufactured morphology of the film in the reflective-type pump-probe experiments, where the films are melted and ablated a little in the center to form a carter-like structure by a laser beam, and the rest of the materials are deposited around the zone. However, the laser fluence in the experiments is too small to ablate the GST films with a thickness of 100 nm; thus, there are still GST materials in Zone A’, and the substrate of silicon is not exposed. The other one, Zone B’, is the microstructure formed on the film, where the phase transition of the films occurs only. In the center of Zone A’, grains with small particles are formed for the pure GST shown in Figure 5a2, while the grains get together into pieces with small particles on them for the N-doped GST shown in Figure 5b2. From Figure 5a3,b3, it can be easy to distinguish the edge of Zone B’ because of variations in electron scattering or resistivity. The edge of the N-doped GST is more obvious than that of the pure GST. There are more and bigger defects around the edge for the N-doped GST. In addition, when nitrogen is doped into GST, there is porosity formation, which results in bigger surface roughness in the as-grown areas. However, Zone B’ becomes smooth after laser irradiation for both the pure GST and the N-doped GST, where small crystalline grains are formed.

**Figure 6:**
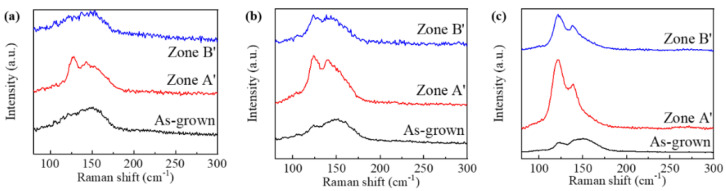
(**a**) Raman spectrum of pure GST in different regions, (**b**) Raman spectrum of 2:1 films in different regions, and (**c**) Raman spectrum of 2:2 films in different regions.

To explore the crystallization evolution induced by the femtosecond laser beam pulse for three kinds of films, as-grown films, Zone A’, and Zone B’, on three kinds of films are measured by the Raman spectrum, as shown in Figure 5. For pure GST films, the as-grown films show a broad peak at about 150 cm^−1^ in Figure 6a, which is related to the stretching vibrations of Te-Te bonds Sb_m_Te_3_ (*m* = 1, 2) pyramidal units and is a characteristic peak for amorphous GST [27,28]. Similarly, there are the same peak for 2:1 and 2:2 films, which can be found in Figure 6b,c, respectively. However, the peak at about 150 cm^−1^ decreases a little because the nitrogen incorporation improves the bond effect, which increases the number of Ge-N bonds. This will suppress crystalline grain growth and enhance thermal stability [29]. In addition, there is another peak at around 125 cm^−1^ for these as-grown GST films. With an increase in nitrogen content, the peak at about 125 cm^−1^ is more obvious due to the distorted octahedral vibration mode [30]. After laser beam irradiation, the peak of 150 cm^−1^ becomes wider, even not obvious in Zone B’ for pure GST films, as shown in Figure 6a, and there is a broad peak of around 125 cm^−1^, which is attributed to the A_1_ mode of GeTe_4-n_Ge_n_ (*n* = 0, 1, 2) corner-sharing tetrahedral units [31]. Moreover, a peak of 108 cm^−1^ can be found, which results from the A_1_ symmetry of GeTe_4-n_Ge_n_ (*n* = 0, 1, 2, 3) tetrahedral units. It is considered a characteristic peak of crystallization [32]. For N-doped GST, the more nitrogen content is, the more obvious the peak of 125 cm^−1^ is in Zone B’. With the increase in the nitrogen content, there are peak shifts from 150 cm^−1^ to 140 cm^−1^, which is ascribed to Te–Te bonds and indicates the segregation of the Te crystalline phase [28], then shifts to 135 cm^−1^, which is attributed to the A1 mode of GeTe_4_-nGen (*n* = 1, 2) tetrahedra and means the crystalline phase [33]. In the ablated Zone A’, the peak at 145 cm^−1^, related to Sb-Te vibrations in Sb_m_Te_3_ (*m* = 1, 2) pyramidal units, is shown as the curve of the pure GST films. There is another peak at about 125 cm^−1^, which is stronger than the peak at 145 cm^−1^. As in Zone B’, there is the same variation of the peak at 125 cm^−1^ and shifts for N-doped GST in Zone A’, and the peak at 125 cm^−1^ is stronger than that in Zone B’. The Raman spectrum profiles of Zone A’ for these films correspond to the over-irradiation reported in the literature [28].

### 3.3. Threshold Evolution Analysis of Irradiation Regions

**Figure 7:**
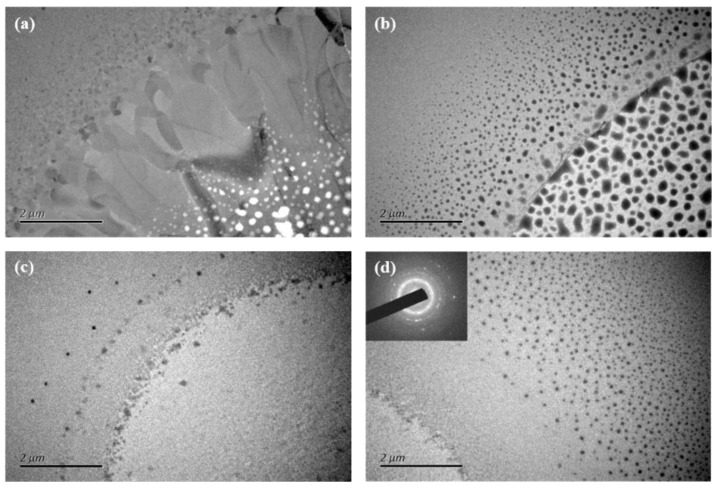
(**a**) TEM images of irradiated pure GST films with a fluence of 0.023 J/cm^2^, (**b**) TEM images of irradiated 2:1 films with a fluence of 0.023 J/cm^2^, (**c**) TEM images of irradiated 2:2 films with a fluence of 0.023 J/cm^2^, (**d**) TEM images of irradiated 2:2 films with a fluence of 0.021 J/cm^2^.

To study the threshold evolution of these films, the crystalline microstructures of the pure GST and different nitrogen contents of GST films irradiated by a femtosecond laser beam are investigated by TEM. To discover the different crystallization processes among these films, the same laser fluence of 0.023 J/cm^2^ is used, which is more than the ablation threshold of these films. As shown in Figure 7a–c, all the films are ablated in the center, while the diameters of the ablated regions become smaller as the nitrogen is doped. It is indicated that the thermal stability is enhanced with the increase in the nitrogen content, which is in agreement with the above analysis in the paper. For the pure GST films, the TEM morphology experiences un-crystallization, solid-state and liquid–solid crystallization, and ablation process in Figure 7a [11]. When the fluence is below the phase transition threshold, the laser-irradiated area is still in an amorphous state. When the fluence is between the phase transition threshold and the melting threshold, there is a nucleation-driven crystallization process in this zone known as the solid-state crystallization area, which finally forms equiaxed nanocrystals [34]. This is because when the femtosecond laser irradiates the materials, the electron is excited, which is analyzed in Section 3.1. This could have an influence on atomic diffusion. Then, the energy would transfer from the electron to the lattice, which would vibrate the covalently bonded backbone of the crystalline lattice [35]. Thus, this results in a phase transition. In this process, the temperature of the lattice is lower than the melting point of the materials. When the fluence increases but is less than the ablation threshold, the zone is under a liquid–solid phase transition process. The temperature of the lattice makes the materials melt, and there is an initial nucleus at the liquid–solid interface, which grows into columnar grain along the radius of the irradiation spot driven by the temperature gradient [36]. When the fluence is greater than the ablation threshold, the materials are removed by the laser beam, which shows the white spots in Figure 7a. However, there are still columnar grains in this zone. The crystallization process of the N-doped GST films is similar to that of pure GST films, which are the four zones mentioned above. For the 2:1 films, equiaxed nanocrystals are formed in the solid-state crystallization area, whose diameter is smaller than that of the pure GST films, as presented in Figure 7b. The nucleus grows a little bigger at the liquid–solid interface but is still smaller than that of the pure GST films. In the ablated zones, there are still equiaxed nanocrystals. In Figure 7c, it is obscure in the irradiated zone for the 2:2 films because of the over ablation by the laser beam, except there are some nanocrystals around the solid-state crystallization area. When the fluence decreases, crystallization characterization can be observed, as shown in Figure 7d. The diameter of the equiaxed nanocrystals is smaller than that of the 2:1 films in the solid-state crystallization area. The nanocrystals in the over-ablation zones still appear obscurely, as proved by the inserted SAED patterns. It can be seen that the crystallization fluence, shape, and size of the nanocrystals will change because of the bond effect.

**Figure 8:**
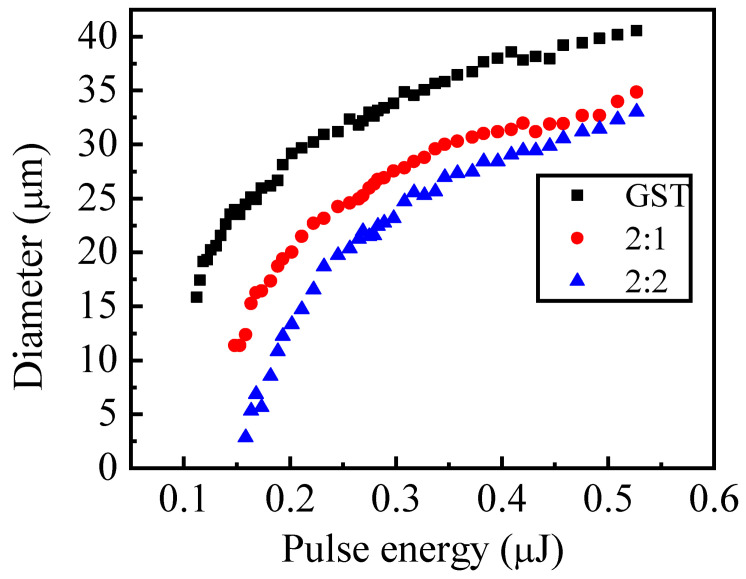
Diameter of different samples ablated by a single laser pulse.

To study the threshold of different kinds of films on the laser fluence, the pure GST and different nitrogen content films are focused through the same lens used in the reflective-type pump-probe experiments by single femtosecond laser pulses. The distance between the focal lens and the sample kept as the focal distance was studied in the experiments. Single-pulse laser beams with different fluence were used to explore the relationship between the laser power and the spot size. As the laser fluence increases, the phase state of the films changes first. Then, the films are ablated. The experiments were carried out three times, and the relation between the laser fluence and the average diameter of the films ablated by a single pulse is shown in Figure 8. 

The phase transition threshold refers to the minimum laser fluence $\varphi_{th}$ to modify the phase state of the materials, which can be derived as [37]

(1) $$D^{2}=2\omega_{0}^{2}\cdot ln(\varphi_{0}/\varphi_{th})$$

where $\omega_{0}$ is the radius of the laser beam at 1/*e*^2^ in the Gaussian spatial beam profile, and *D* is the diameter of the phase transition region versus the maximum laser fluence in front of the surface $\varphi_{0}$, which can be expressed by [37]

(2) $$\varphi_{0}=2E/(\pi \omega_{0}^{2})$$

where *E* is the laser pulse energy. The phase transition threshold $\varphi_{pth}$ and the Gaussian beam radius $\omega_{0}$ can be fit based on the curves in Figure 8 according to Equation (2). The beam radiuses of $\omega_{0}(GST)$ = 31.728 μm, $\omega_{0}(2:1)$ = 31.510 μm, and $\omega_{0}(2:2)$ = 32.420 μm are obtained by the least square fit from the pure GST, 2:1, and 2:2 films, respectively. However, the fit laser beam radius should be the same because of the same conditions as in these experiments; thus, the average beam radius $\omega_{0}$ is calculated as the final value, which is $\omega_{0}=(\omega_{0}(GST)+\omega_{0}(2:1)+\omega_{0}(2:2))/3$ = 31.886 μm. Then, the phase transition thresholds of each kind of film are fit by the least square method again, which are $\varphi_{pth}(GST)$ = 0.005 J/cm^2^, $\varphi_{pth}(2:1)$ = 0.008 J/cm^2^, and $\varphi_{pth}(2:2)$ = 0.01 J/cm^2^.

Compared to different kinds of films, there is a minimum phase transition threshold for pure GST films, while the phase transition threshold of 2:2 films is the highest. This means that it is easy for the pure GST films to crystallize among these kinds of films, while it is hard for 2:2 films from the analysis on the phase transition thresholds. With the increase of nitrogen doping, the phase transition threshold of the doped GST becomes high, crystallization becomes hard, and thermal stability improves. This is consistent with the mention of the bond effect in the paper.

## 4. Conclusions

In this paper, the influences of the element-doped PCM on ultrafast dynamics, crystallization evolution, and thermal stability induced by a femtosecond laser pulse were studied, and a bond effect was demonstrated. For example, the ultrafast dynamics of the pure GST and different nitrogen contents of doped GST films were observed by reflective-type pump–probe experiments. The results indicated that the more nitrogen that was doped, the more free carriers were in the films, and the faster the energy transferred from the free carriers to the lattice of the films. As nitrogen was doped in the GST, the bond effect was enhanced, which suppressed grain growth and improved thermal stability. In addition, the bond effect also influences the microstructure after the ablation of the laser beam. According to the analysis in the paper, the desired performance of PCMs could be gained by modifying the bond effect.

## Data Availability

Not applicable.
